# Probenecid slows disease progression in a murine model of autosomal dominant polycystic kidney disease

**DOI:** 10.14814/phy2.15652

**Published:** 2023-04-06

**Authors:** Sergey N. Arkhipov, D'Anna L. Potter, Regina F. Sultanova, Daria V. Ilatovskaya, Peter C. Harris, Tengis S. Pavlov

**Affiliations:** ^1^ Division of Hypertension and Vascular Research Henry Ford Health Detroit Michigan USA; ^2^ Department of Physiology Wayne State University Detroit Michigan USA; ^3^ Division of Nephrology Medical University of South Carolina Charleston South Carolina USA; ^4^ Department of Physiology, Medical College of Georgia Augusta University Augusta Georgia USA; ^5^ Department of Nephrology and Hypertension, Mayo Clinic Rochester Minnesota USA

## Abstract

Development of autosomal dominant polycystic kidney disease (ADPKD) involves renal epithelial cell abnormalities. Cystic fluid contains a high level of ATP that, among other effects, leads to a reduced reabsorption of electrolytes in cyst‐lining cells, and thus results in cystic fluid accumulation. Earlier, we demonstrated that *Pkd1* ^*RC/RC*^ mice, a hypomorphic model of ADPKD, exhibit increased expression of pannexin‐1, a membrane channel capable of ATP release. In the current study, we found that human ADPKD cystic epithelia have higher pannexin‐1 abundance than normal collecting ducts. We hypothesized that inhibition of pannexin‐1 function with probenecid can be used to attenuate ADPKD development. Renal function in male and female *Pkd1* ^*RC/RC*^ and control mice was monitored between 9 and 20 months of age. To test the therapeutic effects of probenecid (a uricosuric agent and a pannexin‐1 blocker), osmotic minipumps were implanted in male and female *Pkd1* ^*RC/RC*^ mice, and probenecid or vehicle was administered for 42 days until 1 year of age. Probenecid treatment improved glomerular filtration rates and slowed renal cyst formation in male mice (as shown in histopathology). The mechanistic effects of probenecid on sodium reabsorption and fluid transport were tested on polarized mpkCCD_cl4_ cells subjected to short‐circuit current measurements, and in 3D cysts grown in Matrigel. In the mpkCCD_cl4_ epithelial cell line, probenecid elicited higher ENaC currents and attenuated in vitro cyst formation, indicating lower sodium and less fluid retention in the cysts. Our studies open new avenues of research into targeting pannexin‐1 in ADPKD pathology.

## Graphical abstract

Probenecid inhibits pannexin‐mediated ATP release into ADPKD cysts.

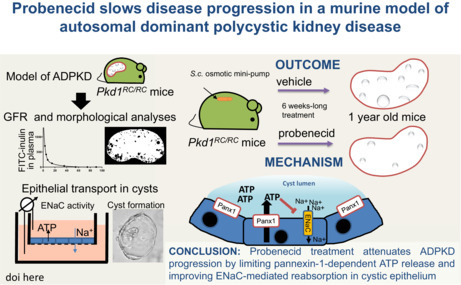

**New and noteworthy**

Pannexin‐1 is a membrane protein, which is permeable to ATP. We found abnormally high expression of pannexin‐1 in renal cysts of ADPKD patients. Pannexin‐1 blockage with probenecid reduced luminal ATP release maintained, the glomerular filtration rate and slowed renal cyst development by improving epithelial transport across cyst epithelium.

## 1 INTRODUCTION

Autosomal dominant polycystic kidney disease (ADPKD) is a monogenic disorder leading to the development of multiple cysts along the nephron. Over the years, cysts grow and replace normal functional tissues, causing an increase in kidney size, tissue damage, and renal insufficiency. The majority of ADPKD cases is caused by mutations in *PKD1* (in 78% of disease pedigrees) or *PKD2* (in 15% of disease pedigrees). Genetic analyses report that the severity of ADPKD is higher in truncating than nontruncating *PKD1* mutations, and the least disease severity is observed in patients with *PKD2* mutations (Cornec‐Le Gall et al., 2013). *PKD1* and *PKD2* encode polycystins, PC1 and PC2, respectively, that are abundant in cilia, endoplasmic reticulum, and plasma membranes, where they regulate the intracellular calcium level (Nauli et al., 2003).

In addition to the role of genetic factors, ADPKD manifestation depends on cellular and tissue reactivity, which counterbalances the pathogenic impact of the mutations. For instance, tolvaptan (the only drug approved by FDA for ADPKD treatment) slows cyst progression by controlling the cytosolic cAMP level (Wang et al., 2005). However, despite the availability of tolvaptan, there is still a strong demand for alternative therapeutic strategies as well as for new molecular targets for pharmacological intervention in ADPKD. One of the understudied pathogenic factors contributing to cyst growth is the abnormal ATP level reported in cystic fluid (Ilatovskaya et al., 2016; Sudarikova et al., 2021). We recently showed that in contrast to normal nephrons, the cystic epithelium exhibits excessive levels of pannexin‐1, a hemichannel capable of ATP release (Arkhipov & Pavlov, 2019). Verschuren et al. (2020) also reported that inhibition of pannexin‐1 with brilliant blue‐FCF led to smaller cysts in the zebrafish *pkd2*‐MO ADPKD model. The ability of pannexin‐1 to mediate extracellular ATP release has been shown in different tissues (Beckel et al., 2015; Cheung et al., 2016; Dahl, 2018; Sang et al., 2019; Taruno, 2018).

Probenecid, employed in this study, is a nonselective blocker of organic anion transport, including transport via pannexin‐1; probenecid is widely used in anti‐gout applications due to attenuation of uric acid reuptake in the nephron (Robbins et al., 2012). Studies from various fields provided evidence that probenecid can be employed as a pannexin‐1 blocker (Silverman et al., 2008; Wei et al., 2021). Here, we show that pannexin‐1 blockage provides therapeutic effects in a mammalian model of ADPKD, caused by a mutation in the *Pkd1* gene. The *Pkd1* ^*RC/RC*^ mouse strain (Hopp et al., 2012) harbors the p.Arg3277Cys variant of *PKD1*, identified in a consanguineous family of French ancestry in the United States. Viable homozygous patients of this family had mild to typical manifestation of cystic disease. Mice, mimicking this human mutation, are viable for at least 1 year as homozygotes and have slow progression of PKD. Due to a panel of traits close to human ADPKD (late onset, sex differences, high blood urea nitrogen (BUN), and predominant (~70%) development of cysts in the collecting ducts (CD) of mature mice, this hypomorphic model of ADPKD is widely used in preclinical studies (Hopp, Catenacci, et al., 2022; Hopp et al., 2015; Hopp, Kleczko, et al., 2022; Jamadar et al., 2021; Pastor‐Soler et al., 2022). Our studies provide the first evidence that pannexin‐1 is a specific target in the epithelium of human cysts and aim to show how probenecid inhibits cyst formation in vivo.

## 2 MATERIALS AND METHODS

### 2.1 Human samples and animals

Depersonalized histological nephrectomy samples from patients with ADKPD and healthy kidney specimens were kindly provided by the Mayo Clinic Translational PKD Center. Ethical treatment of patients is approved by IRB protocol #11‐002357 (Mayo Clinic Cystic Kidney and Liver Disease Biobank). These samples were collected from a female patient with a frameshifting, truncating mutation, c.10797_10800del (p.Val2173Gly), and two males with missense, non‐truncating variants, c.6518T>G (p.Phe3600fs) and c.7702A>G (p.Arg2568Gly) in the *PKD1* gene. The center also provided male and female *Pkd1* ^*RC/RC*^ (in the C57BL/6J background) mice, and a breeding colony was established at the Henry Ford Health System. A group of C57BL/6N (strain code 701) mice was purchased from Charles River Laboratories and used in control experiments. Animals were housed on standard 12:12 h light: dark cycle with water and food (Envigo Teklad 8640) provided ad libitum. Studies were conducted on 9–20 month‐old male and female mice, randomized into treatment groups. All mice were used in compliance with the ARRIVE guidelines, and the studies were approved by Wayne State University/Henry Ford Health institutional animal care and use committees.

### 2.2 Immunohistochemistry

**FIGURE 1:**
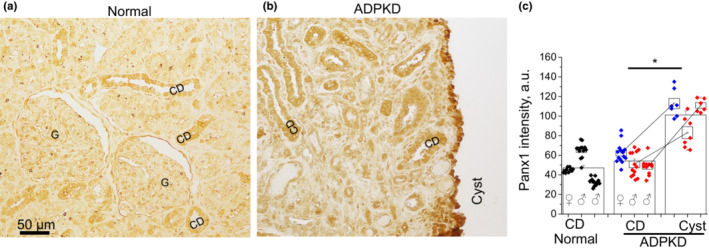
Immunohistochemical staining in human autosomal dominant polycystic kidney disease (ADPKD) biopsy shows higher pannexin‐1 level in the cystic epithelium than in collecting ducts (CD). Representative pannexin‐1 staining in a normal kidney (a) and ADPKD patient with mutations in the *PKD1* gene (b). G, glomerulus. (c) Summary graph of pannexin‐1 abundance. Normal kidneys (one female and two males) are represented by black symbols grouped per each of individuals; comparison CDs versus cysts in three ADPKD patient kidneys is shown as three pairs of values. Blue symbols denote data from the female patient with truncating frameshift mutation, red symbols—two male patients with nontruncating missense mutations. Boxes are mean ± SEM for each individual in the corresponding tissue. Columns are intensity mean ± SEM for each tissue type. *p < 0.05 calculated with unpaired or paired *t*‐test. *N* = 3 patients in each group.

For morphological studies, whole kidney slices were stained with hematoxylin–eosin, and slides were scanned with a high‐resolution Leica Aperio scanner. Cyst area was automatically calculated with the use of Analyze Particles module in the Fiji software package (NIH). Objects with an area exceeding 10,000 μm^2^ and circularity above 0.05 were identified as cysts. Pannexin‐1 protein in human samples was stained with the primary monoclonal or polyclonal antibodies (SAB1403167 and HPA016930, Sigma‐Aldrich). IgG2a kappa isotype control antibodies were obtained from Sigma‐Aldrich (cat#M9144) and tested on normal human kidney biopsy specimens, purchased from Novus Biological (cat#NBP2‐77572). Primary antibodies were used in 1:20 dilution. For secondary staining, we used biotinylated anti‐rabbit antibody 1:200 (BA‐1000, Vector Laboratories) and Streptavidin‐HRP (Dako K0609). Visualization was done with the DAB Substrate (Dako K3468). IHC images were captured at 40× magnification with a Nikon DS‐Ri1 camera interfaced with Nikon Elements software package. The intensity of pannexin‐1 staining was blindly measured with Fiji software: Average signal was measured in regions of interest in CD, cystic epithelia, and background areas. Each point in Figure 1; Figure S1 represents the signal level (0–255 arbitrary unit scale) of an individual CD or cyst after subtraction of background.

### 2.3 Osmotic minipumps

Alzet 2006 was used for continuous drug administration (for 42 days) to 10.5 month‐old *Pkd1* ^*RC/RC*^ and control C57Bl/6N mice. Probenecid (Santa Cruz Biotechnology, 202773) was dissolved in 1 M NaOH and slowly titrated with HCl until pH 7.4. The solution was diluted until it was isosmotic, and the probenecid concentration reached 50 mg/mL. Probenecid or vehicle (saline) was loaded in minipumps and implanted subcutaneously to deliver the drug (180 μg/day). Surgery was performed under isoflurane anesthesia, and Buprenorphine SP was used for post‐surgery analgesia. After treatment, the 12‐month‐old animals were subjected to glomerular filtration rate (GFR) measurements. At the endpoint, blood samples were collected, and the kidneys were flushed by retrograde perfusion with saline. Levels of BUN were measured with the Urea Fluorometric Assay Kit #700620 (Cayman Chemicals).

GFR in conscious mice was studied according to the method previously described by (Rieg, 2013). FITC‐labeled inulin (TdB Consultancy) was dialyzed in saline through a 3.5 kDa membrane (EMD Millipore 71508–3). Mice were anesthetized with isoflurane, inulin was injected into the retro‐orbital sinus at 80 μg/g body weight, and the tail tip was snipped. Mice were placed into individual cages where they gained consciousness within ~2 min. A series of 10 μL blood samples from the tail were collected in heparinized minicapillaries at 3, 5, 7, 10, 15, 35, 55, 75, and 90 min after the injection. After this, plasma FITC‐inulin concentration was measured with a Nanodrop 3300 fluorimeter. Data were fitted with two‐phase exponential decay, according to the two‐compartment model (Sturgeon et al., 1985), and GFR rate was calculated in mL/min/100 g total body weight.

### 2.4 Patch‐clamp electrophysiology

CHO‐K1 cells (CCL‐61, ATCC) were grown, transfected, and prepared for patch‐clamp as reported earlier (Ho et al., 2018). Briefly, 0.5 μg mPanx1 and 0.25 μg mGFP (plasmids) were transfected into the cells and seeded onto glass chips with Lipofectamine 3000 (#MR206795, #TR30007, Origene and #L3000‐008, Invitrogen). One to three days later, currents were acquired and subsequently analyzed with an Axopatch200B amplifier (Axon Instruments) and Bessel filter LPF‐8 at 200 kHz (Warner Instruments), interfaced via a Digidata 1550B to a PC running the pClamp 10.6 suite of software (Axon Instruments). Symmetrical bath and pipette buffer solutions contained (in mM): 25 KCl, 125 NaCl, 1 EGTA, 1 EDTA 10 HEPES, and pH 7.4. For each patch‐clamp experiment, a glass chip with cells was transferred into the chamber with a constant flow of bath solution and placed under the inverted microscope Nikon Ti‐S.

### 2.5 Epithelial cell culture

The mpkCCD_cl4_ cell line, representing epithelial cells of mouse cortical CD, was previously characterized and extensively employed in the studies, focused on Na^+^ transport via ENaC (Bens et al., 1999; Ho et al., 2018). Cells were grown in DMEM:F12 media (10‐090‐CV), containing 2% FBS, 1% insulin‐transferrin‐selenium mix (Gibco 41400‐045), and 1% Penicillin–Streptomycin Glutamine (Gibco 10378‐016). Cells were passaged onto permeable membranes (~0.8 × 10^6^ cells/insert Corning 3450) and grown for 10–12 days until a monolayer with high resistance developed (~1 kΩ·cm^2^). The permeable support system has apical and basolateral chambers separated by a cellular monolayer growing on a permeable 0.22 μm pore membrane. ATP production was stimulated with 60 min of slow rocking. Samples of the media were collected from the apical chamber before and after flow stimulation, and ATP levels were measured with bioluminescent luciferin/luciferase assay kit (Invitrogen #A22066). For trans‐epithelial current measurements, the current was probed with a Volt/Ohm meter (World Precision Instruments) before and after treatment with probenecid. Amiloride (10 μM) was added at the end of the experiment to determine the ENaC‐mediated portion of the current. MpkCCD_cl4_ cells were also used to model cyst development in 3D culture similarly to Mangoo‐Karim and colleagues (1989). Approximately 5000 cells were mixed 1:1 in Matrigel (Corning 356234) and passaged with 10 μM forskolin (Tocris 1099), and vehicle/probenecid/amiloride was added in wells (24 well plate Corning 3598). Cysts were grown for 3 weeks, calibrated images were acquired at 40× magnification with an Olympus IX81 inverted microscope interfaced via Metamorph software, and sizes of the cysts were measured with Fiji software package (NIH) by manual selection of each cyst wall borders.

### 2.6 Statistics

Data are reported as mean ± SEM; *t*‐test or ANOVA was used to compare two or multiple groups, respectively. ANOVA was used with Dunnett's correction in the case of multiple comparisons versus a control group. All other multiple comparisons were corrected with the Tukey test. Regression analyses and comparison were performed using GraphPad Prism 9 and OriginPro 9 software.

## 3 RESULTS

### 3.1 Cyst‐lining epithelium exhibits elevated level of pannexin‐1 in human ADPKD

In order to test the clinical relevance of pannexin‐1 in cystogenesis, we obtained human biopsy samples and stained them with anti‐PANX1 antibodies. The image in Figure 1 depicts the distribution of PANX1 in renal tissues. Since most human cysts originate from the collecting duct system (Verani & Silva, 1988), we compared signal intensity between cysts and CDs of three ADPKD patients and three healthy kidney samples probed with monoclonal antibodies. Nondilated CD in normal and ADPKD kidneys exhibited similar pannexin‐1 signal intensity. However, developed cysts had significantly higher expression of pannexin‐1 than nondilated CDs from the same biopsy sample. This effect was observed in both kinds of mutations: in the patient with a truncating frameshift mutation (blue symbols) and the two patients with different nontruncating missense mutations (red symbols). We also used polyclonal antibodies and data shown in Figure S1a confirm our findings that cystic epithelium expresses higher pannexin‐1 level than CD. Figure S1b shows an IgG isotype negative control for Figure 1. In order to test the mechanistic involvement of pannexin‐1 in cystogenesis in vivo, we performed the next series of experiments in *Pkd1* ^*RC/RC*^ mice.

### 3.2 Age‐dependent changes in GFR in *Pkd1* ^*RC/RC*^ mice

**FIGURE 2:**
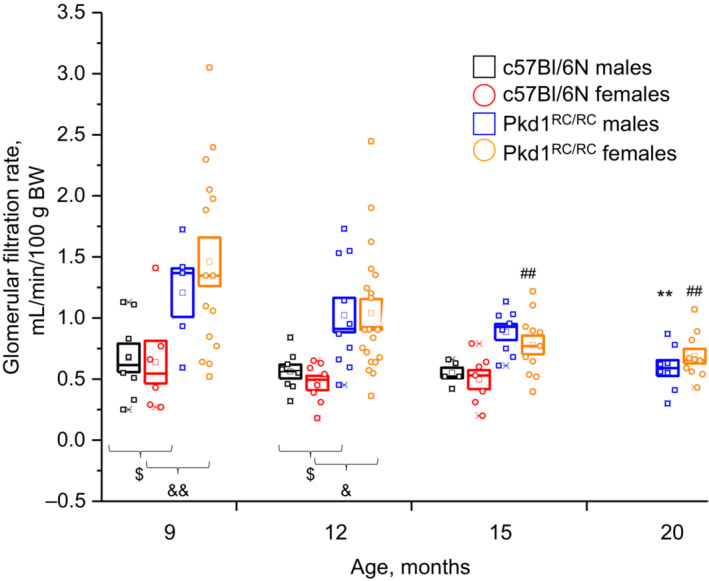
Time course of glomerular filtration rate in *Pkd1* ^*RC/RC*^ and normal C57Bl/6N mice. Plasma inulin clearance after intravenous bolus injection was measured in conscious animals to assess the kidney function with autosomal dominant polycystic kidney disease progression. Individual measurements (squares—males and circles—females) were grouped for mice of 9‐, 12‐, 15‐, and 20‐month ages and reported as mean ± SEM. **p < 0.01 and ^##^ *p* < 0.01 versus 9‐month‐old animals per ANOVA with Dunnett's correction; ^$^ *p* < 0.05, ^&^ *p* < 0.05, ^&&^ *p* < 0.01 per ANOVA with Tukey correction. *N* = 6–20 mice.

Gradual GFR decline is a hallmark of human ADPKD (Chebib & Torres, 2021). Although the RC strains have been widely characterized morphologically, genetically, and metabolically (Arroyo et al., 2021; Hopp et al., 2012, 2015, 2022), GFR data in these mice are still limited. Recently, Pastor‐Soler et al. (2022) reported a positive effect of metformin treatment on GFR measured with transdermal FITC detection in conscious 12‐month‐old male and female *Pkd1* ^*RC/RC*^ mice. Here, we analyzed GFR values in animals from 9 to 20 months of age to determine the relationship between the slow morphological and metabolic progression of the disease and renal filtration. Figure 2 shows the individual GFR readings and mean values for male and female *Pkd1* ^*RC/RC*^ mice in comparison with the non‐ADPKD C57Bl/6N strain. Non‐ADPKD mice exhibited similar GFR in males and females (0.67 ± 0.12 and 0.64 ± 0.17 mL/min/gBW, respectively), which stayed stable until 15 months of age. *Pkd1* ^*RC/RC*^ mice had significantly higher GFR in the beginning of the experiment; then, values gradually decreased. Importantly, GFR reduction occurred faster in females than in males, which is in accordance with earlier reports of more severe morphological and BUN manifestations of ADPKD in females. However, although we have observed *Pkd1* ^*RC/RC*^ mice until 20 months of age, GFR remained in the range comparable with normal C57Bl/6N mice.

### 3.3 Effect of probenecid on ADPKD progression

The high expression of pannexin‐1 in the cystic wall, described above, although an interesting observation, did not provide direct evidence to support the hypothesis that increased pannexin‐1 levels contribute to cystogenesis. In order to test whether pannexin‐1 is a cause of, or a compensatory (or neutral) reaction to, tubular dilation, we performed pharmacological inhibition of pannexin‐1 in *Pkd1* ^*RC/RC*^ males with probenecid.

**FIGURE 3:**
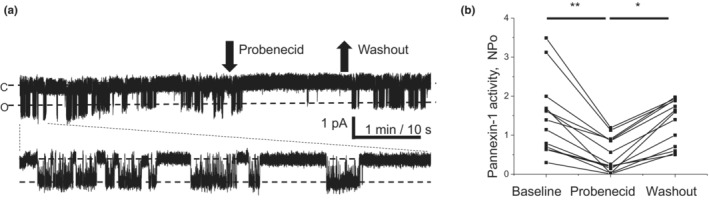
(a) Representative current trace of pannexin‐1 activity recorded from a CHO cell transfected with Panx1 cDNA. Inside‐out activity recorded at −60 mV holding potential, “c” denotes closed and “o” open channel conditions. Arrows illustrate time points of probenecid (75 μM) application and washout. Bottom panel shows the first minute in expanded scale. (b) Summary graph of probenecid and washout effects on pannexin‐1 activity (*p < 0.05; **p < 0.01 ANOVA followed by Tukey correction).

Earlier, an inhibitory effect of probenecid on pannexin‐1‐mediated currents was found with two‐electrode voltage clamp in *Xenopus laevis* oocytes, injected with *Panx1* cRNA (Silverman et al., 2008). To confirm that probenecid is efficient in mammalian cells, we transfected CHO cells with mouse *Panx1* cDNA and recorded channel activity with the patch‐clamp technique. Figure 3 demonstrates pannexin‐1 currents which appear as openings and closings. Probenecid (75 μM) reversibly inhibited pannexin‐1 activity and this validates the use of this drug in mice and a mouse cell line.

**FIGURE 4:**
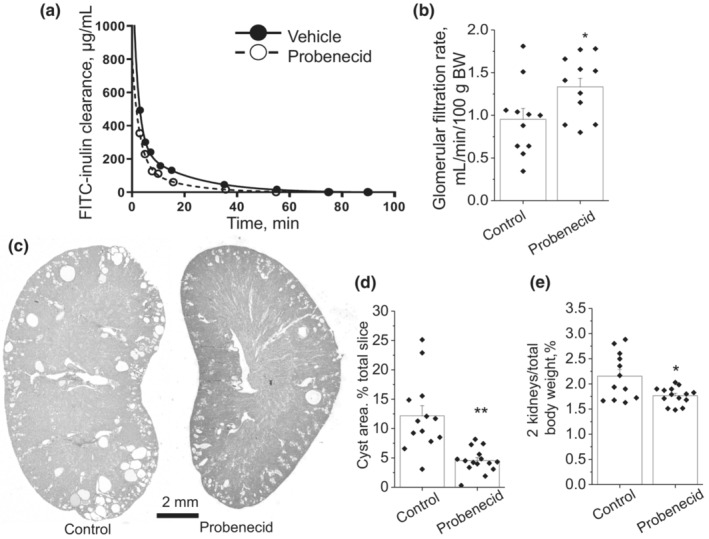
Effect of 6‐week long administration of probenecid on the ADPKD development in the 1‐year‐old male *Pkd1* ^*RC/RC*^ mice. (a) Representative curves of inulin blood plasma show faster clearance in probenecid‐treated versus control mice. (b) Summary graph of glomerular filtration rate (*N* = 11 in each group). (c) Representative transverse kidney sections in the vehicle‐ and probenecid‐treated groups. (d) Summary graph of cyst area percentage related to total slice area. (e) Smaller renal size after the treatment with probenecid, detected with two kidneys / total body weight percentage. *p < 0.05, **p < 0.01 per unpaired *t*‐test. *N* = 13 (vehicle), *N* = 15 (probenecid).

To achieve long‐term administration of the drug without daily disturbance of the animals, we employed drug delivery with minipumps, implanted subcutaneously. After 6 weeks of treatment with probenecid, we measured GFR in 1‐year‐old mice. Typical FITC‐inulin clearance curves in vehicle‐ or probenecid‐treated mice and the summary graph shown in Figures 4a,b indicate that probenecid significantly improved renal filtration function. Morphological data in Figures 4c–e illustrate that probenecid also reduced cyst size and renal hypertrophy. The ratio of the cystic area to the total kidney area was significantly lower in the group of mice treated with probenecid versus the control group (4.5% ± 0.5% vs. 12.2% ± 1.7%). Kidney to body weight ratio was 2.15% ± 0.13% in the control and 1.76% ± 0.05% in the probenecid groups. Therefore, a 6‐week‐long treatment with probenecid was beneficial in 1‐year‐old male *Pkd1* ^*RC/RC*^ mice.

**FIGURE 5:**
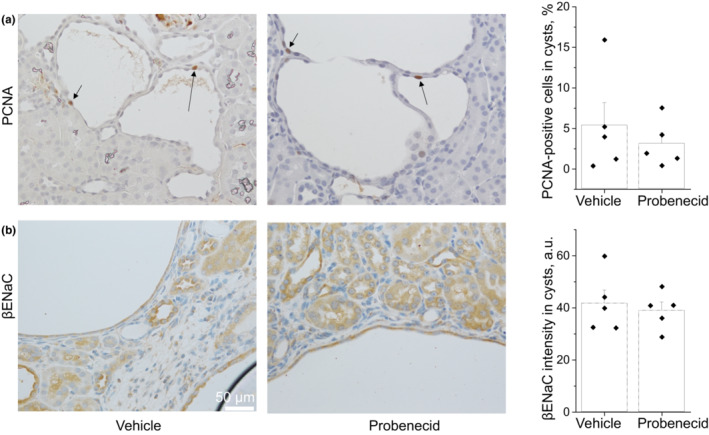
Immunohistochemical staining for PCNA and βENaC in the kidneys after treatment with probenecid. (a) PCNA staining (brown) in nuclei is a marker of proliferating cells. (b) Abundance of βENaC in renal tissues. Markers on the summary graphs represent average value in each kidney; columns are mean ± SEM in the groups. *N* = 5 mice in each group.

Cyst development depends on different factors and we tested whether proliferative activity of cyst‐lining cells was reduced by probenecid treatment. We performed immunohistochemical analysis against proliferating cell nuclear antigen PCNA in the kidneys after vehicle and probenecid treatments. Figure 5a presents kidney sections where some nuclei of the cyst‐lining cells are stained brown (arrows) to indicate the G1/S phase of the cell cycle. Amounts of proliferating cells in vehicle and probenecid‐treated males were similar. We also investigated whether probenecid affected ENaC expression in cyst‐lining cells. IHC experiments detecting βENaC revealed similar ENaC abundance between the groups (Figure 5b). Although protein abundance is a part of ENaC regulation, channel open probability (P_o_) also plays a critical role in its regulation; ATP, to a large extent, affects P_o_ of ENaC (Pochynyuk et al., 2010). To better address the effect of probenecid on ENaC activity, we performed experiments in live immortalized epithelial cells as reported in the sections below.

The effect of probenecid was tested in female *Pkd1* ^*RC/RC*^ mice following the same protocol (Figure S2). However, cystic index was not significantly different in 12‐month‐old females treated with probenecid or vehicle. Similarly, probenecid did not affect kidney weight/TBW ratio or GFR in the females. In a control group of 12‐month‐old male C57Bl/6N mice, probenecid treatment had no effect on GFR (Figure S2d).

**FIGURE 6:**
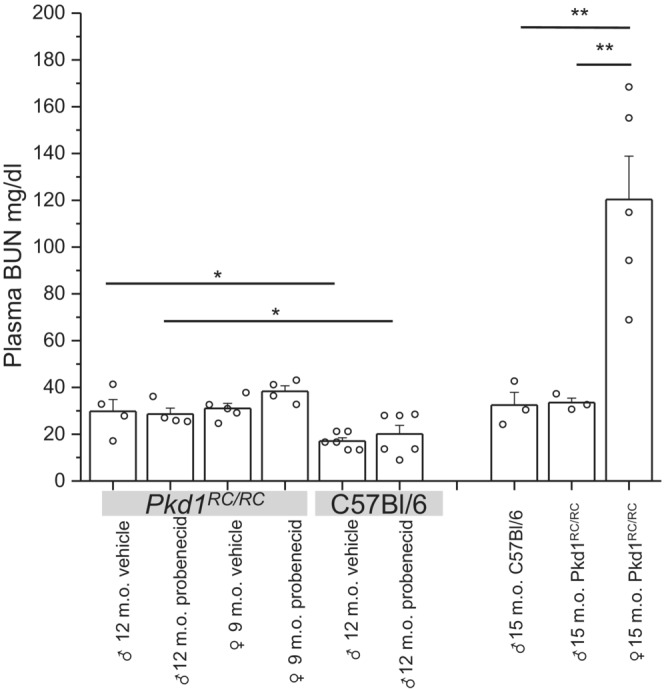
Lack of effect of probenecid on blood urea nitrogen (BUN) level. Blood was collected in the end of treatment from *Pkd1* ^*RC/RC*^ and C57Bl/6N mice. For comparison, aged 15 months old mice were used as control. *p < 0.05; **p < 0.01 ANOVA with Tukey correction.

Since probenecid is a uricosuric agent, we tested how treatment with this drug affected BUN level in *Pkd1* ^*RC/RC*^ and non‐cystic C57Bl/6N mice (Figure 6). Tests revealed that in the vehicle‐treated groups, 12‐month‐old *Pkd1* ^*RC/RC*^ males and 9‐month‐old *Pkd1* ^*RC/RC*^ females had similar BUN levels, which were moderately but significantly higher than in 12‐month‐old non‐cystic C57 males. Therefore, probenecid treatment did not significantly affect nitrogen balance in these three groups. For comparison, 15‐month‐old female *Pkd1* ^*RC/RC*^ mice developed severe azotemia with BUN over 120 mg/dL whereas age‐matched male *Pkd1* ^*RC/RC*^ and C57Bl/N6 mice had BUN ~30 mg/dL.

### 3.4 Mechanism of action of probenecid on cystogenesis

**FIGURE 7:**
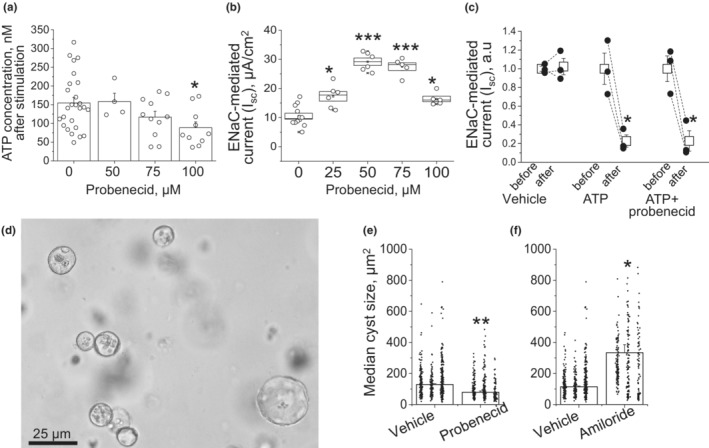
Effects of probenecid on sodium and fluid transport in mpkCCD_cl4_ cells. (a) Accumulation of ATP in the apical chamber after 1‐h mechanical stimulation with rocking and treatment with vehicle and various concentrations of probenecid. (b) Dose‐dependent effect of probenecid on ENaC‐mediated sodium reabsorption. Amiloride‐sensitive current was measured with an epithelial Volt/Ohm meter after 8‐h‐long treatment with probenecid. (c) Effect of apical application of 100 μM ATP on ENaC‐depended I_sc_ without probenecid and in the presence of the drug. (d) mpkCCD_cl4_ cells grown in 3D culture in the presence of forskolin form cysts. (e) Median cyst size in the control‐ and probenecid‐treated wells on summary graph. (f) Cyst growth in Matrigel in presence of 10 μM amiloride. Each dot represents one cyst size, grouped by wells. Columns are mean ± SEM of the three medians in the wells treated with vehicle, probenecid, or amiloride. *p < 0.05 per unpaired *t*‐test.

Abnormal secretion of ATP to the cystic lumen is recognized as a major pathogenic factor (Sudarikova et al., 2021). To determine how probenecid mechanistically impacts cell functions relevant to in vivo observations, we used immortalized renal epithelial collecting duct cells. We studied apical ATP release in mpkCCD_cl4_ cells stimulated by shear stress (1‐h rocking) with or without an application of probenecid. The cells form a polarized highly differentiated monolayer suitable for studies of epithelial physiology (Pavlov et al., 2009). Mechanical stimulation led to accumulation of ATP in the apical chamber from 93 ± 7 to 154 ± 14 nM (*p* < 0.0001, according to Dunnett's test; *n* = 26), whereas pretreatment with probenecid blunted this effect (Figure 7a).

We next studied the effect of probenecid on water‐electrolyte transport across renal epithelia. ATP is known to suppress the activity of ENaC, which is the limiting step for sodium reabsorption in the distal nephron (Toney et al., 2012). Along with the increased chloride secretion shown in cystic epithelia by many groups (Cabrita et al., 2020; Schwiebert et al., 2002; Wallace et al., 1996), lower ENaC activity can lead to accumulation of fluid in the cysts. We hypothesized that probenecid augments ENaC‐mediated sodium uptake from the lumen by limiting ATP release. ENaC‐dependent sodium uptake across mpkCCD_cl4_ epithelial monolayer grown on permeable support was measured before and 8 h after addition of probenecid. At the end of the experiment, 10 μM amiloride was applied to calculate ENaC‐specific trans‐epithelial current. Figure 7b demonstrates a bell‐shaped dose‐dependent upregulation of ENaC activity by probenecid. In comparison with the 1‐h‐long Figure 7a experiment, a lower dose of 50 μM was the most effective when cells were treated for 8 h. The next experiment (Figure 7c) aimed to test whether ATP application overrides pretreatment with 50 μM probenecid. Apical application of ATP reduced ENaC activity in accordance with earlier reports (Pochynyuk et al., 2010; Thomas et al., 2001). This effect was preserved in cells pretreated with probenecid, and thus, we conclude that low luminal ATP due to pannexin‐1 blockage does not affect purinergic reception and signaling in the treated cells.

In the next experiment, we used an in vitro model of cystogenesis and aimed to study how improved ENaC activity or other ATP‐dependent mechanisms affect ADPKD progression. To address this, mpkCCD_cl4_ cells were grown in Matrigel in the presence of 10 μM forskolin. Forskolin‐induced cAMP upregulation promoted formation of three‐dimensional cysts from growing renal epithelial cells (Figure 7d). Wells incubated with probenecid exhibited formation of smaller cysts versus vehicle‐treated groups (Figure 7e). In contrast, 10 μM amiloride significantly increased cyst size indicating that proper sodium uptake is a necessary anti‐cystogenic factor (Figure 7f). We conclude that probenecid limits ATP release via pannexin‐1, thus attenuating the ATP‐dependent decrease in sodium transport from the cystic space.

## 4 DISCUSSION

The beneficial effects of probenecid on attenuation of renal cysts' progression in *Pkd1* ^*RC/RC*^ mice demonstrated the potential significance of PANX1 hemichannel as a drug target. Shum and colleagues reported that PANX1 was preferentially enriched at the apical membrane of polarized MDCK cells grown as monolayer sheets or cyst‐like spheroids (Shum et al., 2019). We demonstrated that pannexin‐1 is abundant in *Pkd1* ^*RC/RC*^ mouse cyst epithelium (Arkhipov & Pavlov, 2019). Later, Verschuren et al. (2020) confirmed that cystogenesis is mediated by pannexin‐dependent ATP release in a zebrafish model of ADPKD. The authors also found that BB‐FCF, a nonselective pannexin‐1 inhibitor, reduced cyst development in *pkd2* zebrafish morphants. Mechanisms of the BB‐FCF effect were mediated by a reduction in ATP release in response to shear stress as found in wild type and *Panx1* ^*−/−*^ mDCT cells. Here, we tested the effect of pannexin‐1 inhibition by probenecid in a mammalian model of ADPKD. Using an immunohistochemical approach, we confirmed the abnormal upregulation of pannexin‐1 level in human ADPKD cysts, supporting the notion that this protein is a potential therapeutic target in ADPKD.

In the current experimental study, we used *Pkd1* ^*RC/RC*^ mice, whose ADPKD pathomorphology has been well described, although there is a lack of physiological data obtained from live animals. We provide data on a slow age‐dependent GFR decline in males and females. However, this strain exhibits overall higher GFR than normal noncystic mice, indicating that the phenotype of this model also reflects renal hemodynamic changes. The nature of hyperfiltration in *Pkd1* ^*RC/RC*^ mice is unknown. Possibly, the global *RC* mutation affects vasomotor reactions of the renal arterioles due to a role of polycystin‐1 in the endothelium. Of note, we used C57Bl/6N mice as a control mouse strain; *Pkd1* ^*RC/RC*^ mice have C57Bl/6J genetic background. Both genetic and renal hemodynamic origins of hyperfiltration in ADPKD would be an important topic of future investigations. The sex differences in ADPKD, reported earlier in the cystogenesis and BUN progression in *Pkd1* ^*RC/RC*^ mice (Arroyo et al., 2021; Hopp et al., 2012), were further supported here by faster GFR decline in females than males. Earlier publications (Pastor‐Soler et al., 2022; Zhang et al., 2019) focused on *Pkd1* ^*RC/R*C^ mice with the 129S6 SvEv and C57BL/6J hybrid background, and *Pkd2* ^*WS25/−*^ models of PKD, reported that GFR values were around 0.7–0.75 mL/min/100 gBW in 12‐ and 9‐month‐old conscious mice, respectively. Our data showed higher GFR (over 1 mL/min/100 g) at this age indicating that the C57BL/6J RC strain has the slowest disease progression among the aforementioned hypomorphic models in accordance with the earlier publication (Arroyo et al., 2021).

Treatment with probenecid was beneficial for both GFR and cyst growth parameters in male mice. The effect on GFR occurred only when cyst size was reduced by probenecid, whereas the drug had no effect either on GFR or on kidney morphology in the RC females and C57Bl/6N control males. This indicates that inhibition of cyst development causes higher GFR rather than direct effects of probenecid on vasomotor properties of renal vasculature.

In females, no significant effect was observed after probenecid administration. Probably, cysts in males are still developing and therefore are sensitive to the treatment by the age of 12 months whereas females (which have a more severe phenotype) have already formed cysts, indicating that proper timing of probenecid intervention is important to target certain phases of cystogenesis. Long‐term subcutaneous administration of probenecid with osmotic pumps resolved a series of experimental limitations. That includes a continuous delivery of the drug versus multiple daily injections over 6 weeks. The use of minipumps allowed us to avoid drug delivery with drinking water: in clinical practice, due to low absorption in GI tract, the dose of oral pills exceed 0.5–1 g (Robbins et al., 2012). The poor direct solubility of probenecid in water, and nonpreferable delivery of the drug, dissolved in toxic dimethyl sulfoxide, could be a problem in the use of the minipumps for drug delivery as well. However, when we dissolved the drug with sodium hydroxide and slowly titrated it with hydrogen chloride to achieve the neutral pH range, such aqueous solution of probenecid was stable. It should be emphasized that the age of the mice was likely a contributing factor to the efficacy of probenecid. Hopp et al. (2012) described that although cysts of proximal origin prevail in young *Pkd1* ^*RC/RC*^ mice, CD cysts become the major manifestation of the disease in adults. Since previous publications indicate that distal nephron cysts would be more sensitive to pannexin‐1 blockage (Arkhipov & Pavlov, 2019; Arkhipov, et al., 2019), we tested the effects of probenecid in 1‐year‐old mice. Importantly, in our study a notable effect of probenecid was only observed in males, whereas in females, the drug only exhibited a nonsignificant trend to lower the cystic index. We interpret this limitation such as that in the females the pharmacological intervention was done too late when cysts were already formed due to faster development of ADPKD. Earlier Hopp and colleagues described a significant sex difference starting at 9 months, with females having more severe disease (Hopp et al., 2015).

The distal nephron, located downstream from the macula densa, is responsible for final urine concentration. Water and sodium reabsorption in the distal nephron is tightly regulated by vasopressin and the renin‐angiotensin‐aldosterone system, whose major effectors are aquaporin‐2 and ENaC, respectively. Blockage of vasopressin receptors is the mechanism of action of tolvaptan, the only FDA‐approved anti‐ADPKD drug (Chebib et al., 2018). According to the classification by Perrone, based on low sodium content, elevated potential difference, short‐circuit current, and conductance, the cysts can be divided into the proximal (nongradient) and distal ones (gradient) (Perrone, 1985). As we demonstrated earlier in ARPKD (PCK rat model), although cysts retain the ENaC‐dependent transport, its intensity is significantly lower than in the normal non‐dilated CDs and slows salt and fluid absorption from the cystic space (Pavlov et al., 2015). Inhibition of extracellular ATP release by CCD cells treated with probenecid (Arkhipov & Pavlov, 2019) allowed us to identify that increased ENaC activity and improved fluid transport across 2D and 3D structures of mpkCCD_cl4_ cells are likely the mechanism of the anti‐cystogenic action of probenecid.

We would like to acknowledge some limitations of the presented study and discuss future directions. Although mature Pkd1^RC/RC^ mice exhibit large cysts and high BUN levels (over 100 mg/dL), this is a slow progressing model of ADPKD. Thus, by 1 year of age BUN stays below 35–40 mg/dL in these mice (normal range in humans: 6–24 mg/dL). Surprisingly, this model preserves glomerular function: GFR in *Pkd1* ^*RC/RC*^ mice declines with aging but stays higher than in age‐matched normal C57Bl/6 mice. The earlier characterization of the model reveals a hypomorphic phenotype (measured as total kidney volume, cyst indices, and BUN level) but GFR has not been reported. Our data suggest a limited suitability of the model if an investigation is focused on GFR decline in ADPKD. For studying the effects on GFR in ADPKD, perhaps, faster developing models should be used. For instance, a recently developed *Pkd1* ^*RC/RC*^ strain in a Balb/cJ or 129S6 genetic backgrounds have more aggressive disease progression with higher immune cells infiltration, fibrosis, and other histopathological parameters (Arroyo et al., 2021). Comparison of GFR dynamics between RC strains with different backgrounds is a subject of future studies.

Another limitation of our study is high variability of GFR in polycystic mice in the first year. This effect may be due to different disease progression in individual animals when some mice still maintain high glomerular function and others exhibit GFR decline. However, of note, GFR uniformly dropped during the advanced stages of ADPKD.

Pharmacological characterization of pannexin‐1 currents expressed in mammalian cells showed that probenecid is not the most potent PANX1 inhibitor: carbenoxolone and DIDS have significantly lower IC_50_ (Ma et al., 2009). However, these two compounds are specific 11β‐hydroxysteroid dehydrogenase and chloride‐bicarbonate exchanger inhibitors, respectively. Both proteins are pivotal for normal nephron function, and we avoided using them because nonspecific data would be hard to interpret. Another potential mechanism of the observed beneficial effect of probenecid is its agonism for TRPV channels. Zaika et al. (2013) reported that the development of cysts in ARPKD (PCK rat) was accompanied by a loss of TRPV4 expression, required for proper calcium signaling. Treatment with GSK1016790A, a selective TRPV4 activator, inhibited cyst growth in PCK rats. Moreover, probenecid can also inhibit the uptake of oxalate via organic anion transporters (Koul et al., 1994), and recently, calcium oxalate crystal deposition was recognized as a mechanism of cyst growth initiation and activation of PKD‐associated signaling pathways (Torres et al., 2019). Although we have not found effects of probenecid on urea transport, reduced calcium oxalate formation might mediate the reported beneficial effect of probenecid treatment.

Pannexin‐1 activity depends on various regulators such as cytoplasmic calcium and purinergic receptors (Locovei et al., 2006, 2007). Earlier, we demonstrated a shift toward P2X_4_ and P2X_7_ receptors' signaling in the cystic epithelia of PCK rats (Palygin et al., 2018). Recent findings further highlighted a more critical role of P2X_7_ over P2X_4_ in ARPKD cysts progression (Arkhipov et al., 2019; Xu et al., 2022); the role of pannexin‐P2X interaction in ADPKD development remains to be studied. Although our results indicate that pannexin‐1 is a promising target in ADPKD treatment, there is a need for more specific pharmacological agents targeting this channel. Growing evidence of the high importance of this protein in other diseases (cancer, central nervous system disorders, inflammatory cell regulation, and vascular diseases) clearly shows the need to design new and specific molecules for inhibiting pannexin‐1.

## AUTHOR CONTRIBUTIONS

**Sergey N. Arkhipov** and **Tengis S. Pavlov** contributed to the draft, writing, study design, and data analysis. **Sergey N. Arkhipov**, **D'Anna L. Potter**, **Regina F. Sultanova**, **Daria V. Ilatovskaya**, and **Tengis S. Pavlov** conducted experiments. **Daria V. Ilatovskaya** and **Peter C. Harris** provided objects of research and advisory support. All authors approved the final version of the manuscript, declare no conflicts of interest, financial, or otherwise, and would like to thank the Augusta University Center for Writing Excellent (Hannah Soblo, PhD, writing consultant) for professional writing feedback and grammar edits. There were no foreign (non‐US) funding sources, foreign in‐kind contributions, or COIs associated with this study.

## FUNDING INFORMATION

This research made possible by funding from the American Society of Nephrology Carl W. Gottschalk Award, NIH DK123266 (TSP), DK090728, DK058816 (PCH), DK105160, and HL148114 (DVI).

## ETHICS STATEMENT

Ethical conductance of research is approved by Mayo Clinic IRB protocol 11‐002357 and Wayne State University IACUC protocol 20‐10‐2798.

## Supporting information


Figure S1.

Figure S2.
