# Supplementary material for: Probenecid slows disease progression in a murine model of autosomal dominant polycystic kidney disease
Source: Physiol Rep. 2023 Apr 6;11(7):e15652. doi: 10.14814/phy2.15652 (PMC10079433; doi:10.14814/phy2.15652)
Supplement: Supplementary file 1 — Figure S1. [file PHY2-11-e15652-s001.pptx]

## Slide 1
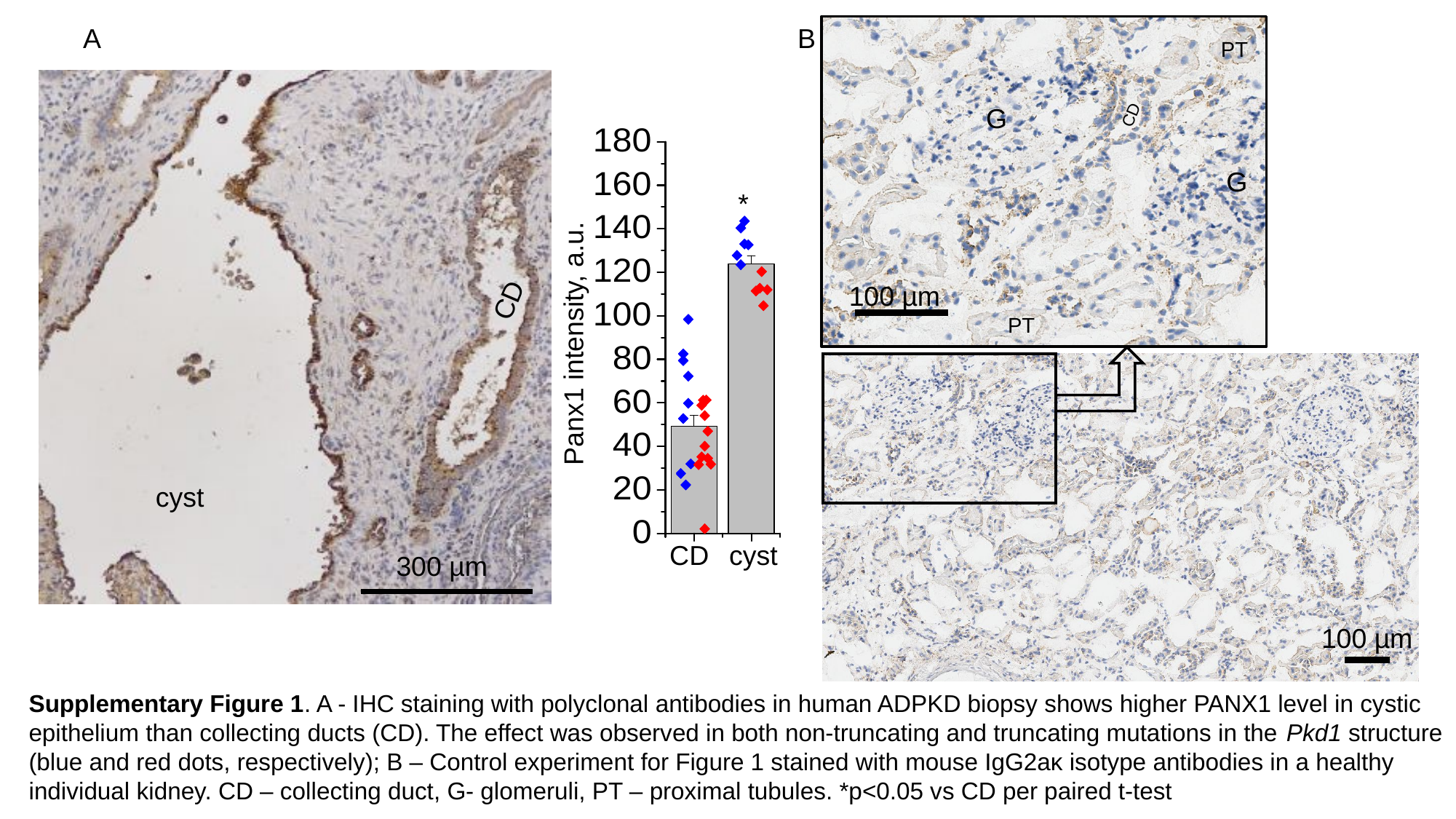

A
B
PT
100 µm
B
CD
cyst
300 µm
G
CD
G
*
100 µm
PT
Panx1 intensity, a.u.
cyst
CD
100 µm
Supplementary Figure 1. A - IHC staining with polyclonal antibodies in human ADPKD biopsy shows higher PANX1 level in cystic epithelium than collecting ducts (CD). The effect was observed in both non-truncating and truncating mutations in the Pkd1 structure (blue and red dots, respectively); B – Control experiment for Figure 1 stained with mouse IgG2aκ isotype antibodies in a healthy individual kidney. CD – collecting duct, G- glomeruli, PT – proximal tubules. *p<0.05 vs CD per paired t-test
