# Supplementary material for: Probenecid slows disease progression in a murine model of autosomal dominant polycystic kidney disease
Source: Physiol Rep. 2023 Apr 6;11(7):e15652. doi: 10.14814/phy2.15652 (PMC10079433; doi:10.14814/phy2.15652)
Supplement: Supplementary file 2 — Figure S2. [file PHY2-11-e15652-s002.pptx]

## Slide 1
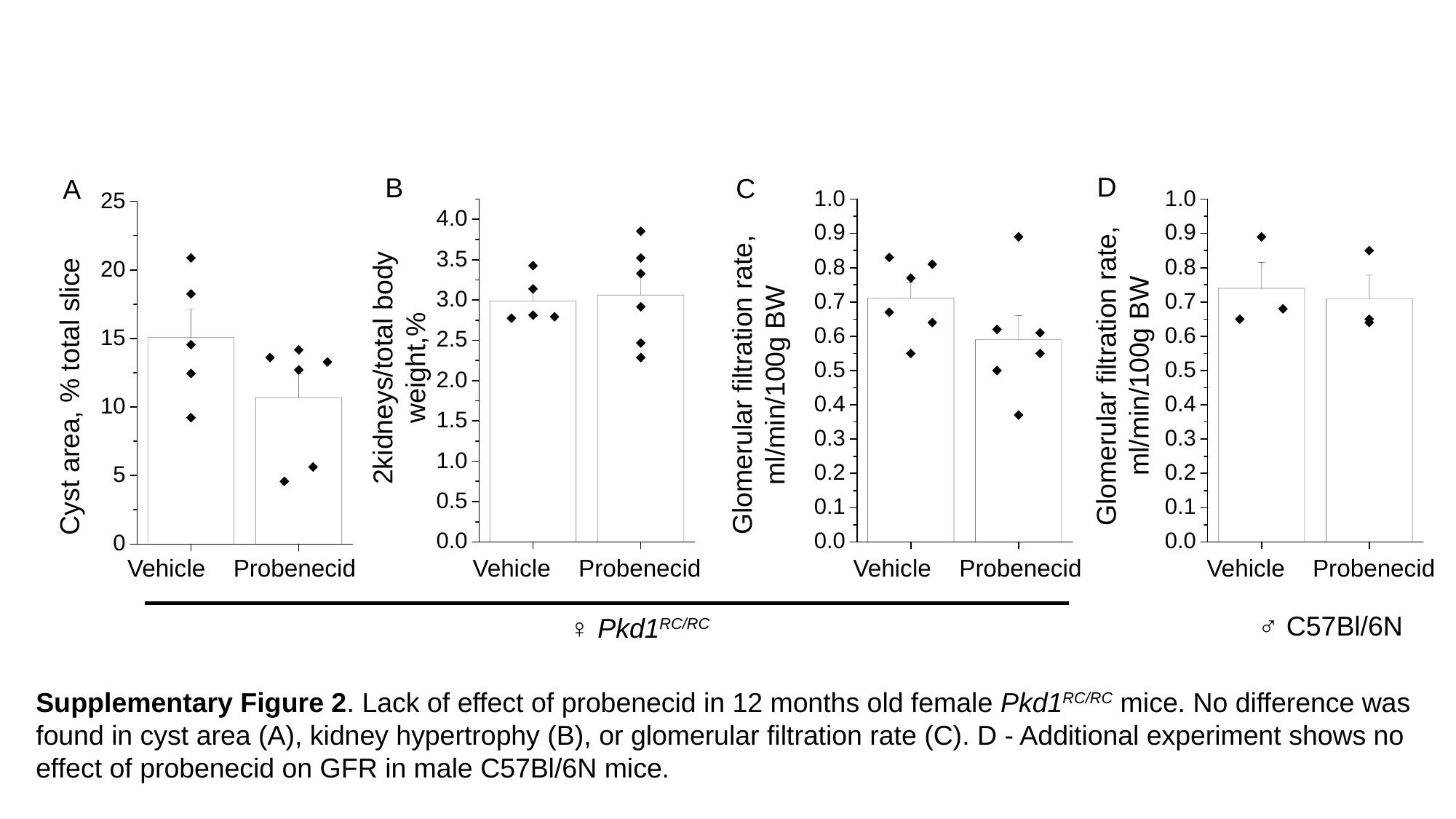

D
B
C
A
2kidneys/total body weight,%
Glomerular filtration rate,
ml/min/100g BW
Glomerular filtration rate,
ml/min/100g BW
Cyst area, % total slice
Vehicle
Probenecid
Vehicle
Probenecid
Vehicle
Probenecid
Vehicle
Probenecid
♂ C57Bl/6N
♀ Pkd1RC/RC
Supplementary Figure 2. Lack of effect of probenecid in 12 months old female Pkd1RC/RC mice. No difference was found in cyst area (A), kidney hypertrophy (B), or glomerular filtration rate (C). D - Additional experiment shows no effect of probenecid on GFR in male C57Bl/6N mice.
